# Phase 3 evaluation of an innovative simple molecular test for the diagnosis of malaria in different endemic and health settings in sub-Saharan Africa (DIAGMAL)

**DOI:** 10.1371/journal.pone.0272847

**Published:** 2022-09-01

**Authors:** Francois Kiemde, Halidou Tinto, Jane Carter, Toussaint Rouamba, Daniel Valia, Lesong Conteh, Elisa Sicuri, Bryony Simmons, Bakri Nour, Davis Mumbengegwi, Asrat Hailu, Stephen Munene, Albadawi Talha, Mulugeta Aemero, Paul Meakin, René Paulussen, Scott Page, Norbert van Dijk, Petra Mens, Henk Schallig

**Affiliations:** 1 Clinical Research Unit of Nanoro, Nanoro, Burkina Faso; 2 Amref Health Africa, Nairobi, Kenya; 3 London School of Economics and Political Science, London, United Kingdom; 4 ISGlobal, Barcelona, Spain; 5 Blue Nile National Institute for Communicable Diseases, University of Gezira, Wad Medani, Sudan; 6 University of Namibia, Windhoek, Namibia; 7 University of Addis Ababa, Addis Ababa, Ethiopia; 8 Jouf University, Sakakah, Saudi Arabia; 9 Gondar University, Gondar, Ethiopia; 10 Innova Partnerships, St Fillans, United Kingdom; 11 Mondial Diagnostics, Amsterdam, The Netherlands; 12 Abingdon Health, York, United Kingdom; 13 Amsterdam Institute for Infection and Immunology, Infectious Diseases Programme, Amsterdam, The Netherlands; 14 Amsterdam University Medical Centres, Academic Medical Centre at the University of Amsterdam, Amsterdam, The Netherlands; Public Library of Science, UNITED KINGDOM

## Abstract

**Background:**

Rapid Diagnostic Tests (RDTs) have become the cornerstone for the management of malaria in many endemic settings, but their use is constrained for several reasons: (i) persistent malaria antigen (histidine-rich protein 2; HRP2) leading to false positive test results; (ii) *hrp2* deletions leading to false negative *Pf*HRP2 results; and (iii) limited sensitivity with a detection threshold of around 100 parasites/μl blood (*p*LDH- and HRP2-based) leading to false negative tests. Microscopy is still the gold standard for malaria diagnosis, and allows for species determination and quantitation, but requires trained microscopists, maintained microscopes and has detection limit issues. Consequently, there is a pressing need to develop and evaluate more sensitive and accurate diagnostic tests. To address this need we have developed a direct on blood mini PCR-NALFIA test that combines the benefits of molecular biology with low infrastructural requirements and extensive training.

**Methods:**

This is a Phase 3 diagnostic evaluation in 5 African countries. Study sites (Sudan, Ethiopia, Burkina, Kenya and Namibia) were selected to ensure wide geographical coverage of Africa and to address various malaria epidemiological contexts ranging from high transmission to near elimination settings with different clinical scenarios and diagnostic challenges. Study participants will be enrolled at the study health facilities after obtaining written informed consent. Diagnostic accuracy will be assessed following the WHO/TDR guidelines for the evaluation of diagnostics and reported according to STARD principles. Due to the lack of a 100% specific and sensitive standard diagnostic test for malaria, the sensitivity and specificity of the new test will be compared to the available diagnostic practices in place at the selected sites and to quantitative PCR as the reference test.

**Discussion:**

This phase 3 study is designed to validate the clinical performance and feasibility of implementing a new diagnostic tool for the detection of malaria in real clinical settings. If successful, the proposed technology will improve the diagnosis of malaria. Enrolment started in November 2022 (Kenya) with assessment of long term outcome to be completed by 2023 at all recruitment sites.

**Trial registration:**

Pan African Clinical Trial Registry (www.pactr.org) **PACTR202202766889963** on 01/02/2022 and ISCRTN (www.isrctn.com/) **ISRCTN13334317** on 22/02/2022.

## Background

### Background and rationale {6a}

Malaria in humans is a life-threatening disease caused by single-cell (protozoan) parasites of the genus *Plasmodium*: *P*. *falciparum*, *P*. *vivax*, *P*. *ovale*, *P*. *malariae* and *P*. *knowlesi*. Malaria is a major global public health problem in tropical and sub-tropical regions with approximately half the world’s population at risk. In 2020, about 219 million malaria cases and nearly 450,000 deaths were reported worldwide. The majority of cases (92%) occurred in sub-Saharan Africa (SSA) [1]. Malaria management has been greatly improved due to wide implementation of World Health Organization (WHO) recommendations that all suspected malaria cases should have a parasite-based diagnosis prior to initiating treatment with artemisinin-based combination therapy (ACT) [2]. Malaria parasite specific antigen-detecting rapid diagnostic tests (RDTs) are considered one of the tools to manage febrile illness in malaria endemic settings [1]. The short time needed to obtain a diagnostic result, simple read-out, easy performance by non-technical staff, and relatively low costs have contributed to the acceptance of RDTs in differentiating malaria from other causes of fever [3]. Most commonly used RDTs are *Plasmodium falciparum* histidine-rich protein 2 (*Pf*HRP2)-based RDTs, specific for *Plasmodium falciparum* only, and *Plasmodium* lactate dehydrogenase (*p*LDH)-based RDTs that detect all *Plasmodium* species, or combination tests with two test lines (a *P*. *falciparum*-specific line and an all *Plasmodium* test line) [4, 5].

However, the implementation of RDTs is constrained by persisting *Pf*HRP2 after successful treatment, which may lead to false positive test results [6–8]. Furthermore, studies report false positive results with *Pf*HRP2-based RDTs particularly in seasonal malaria transmission settings or under harsh environmental conditions [9, 10]. Of greater concern are false negative *Pf*HRP2-RDT results increasingly reported from malaria endemic regions due to *hrp2* deletions in the *Plasmodium* genome [11–13]. In these settings, a HRP2-based RDT will be negative, even if a *P*. *falciparum* infection is present. Finally, RDTs (*p*LDH-based in particular) have sensitivity and stability limitations, also resulting in false negative results [5, 14]. This is particularly evident at low parasitaemias in near malaria elimination settings [15, 16].

Although malaria microscopy remains the standard for clinical diagnosis, the limit of detection (LoD) of malaria parasites by expert microscopy (which is as low as 10 parasites/μl) could miss very low parasitaemias. The LoD is highly dependent on the quality of the equipment, the microscopic reading technique, the experience of the reader and the daily workload [17].

In this context, there is a pressing need to develop more sensitive and accurate malaria diagnostic tests that circumvent the limitations of RDTs and microscopy. In general, molecular biology-based diagnostic tests are considered to be the most accurate methods for detecting malaria infections, irrespective of low parasite densities or *hrp2* deletions [18–20]. Several molecular methods have been developed, including conventional and real-time polymerase chain reaction (PCR), nucleic acid sequence based amplification or loop mediated amplification [19]. These platforms are highly sensitive and some allow species differentiation and parasite quantitation. These formats would be ideal for patient management, but their relative complexity hampers their use as near point of care (PoC) tests. In particular, sample processing (DNA extraction) and complicated read-out systems (gel electrophoresis with UV reading or computerised systems) hamper wide implementation [19].

To circumvent these challenges, the DIAGMAL consortium has developed a simple molecular diagnostic platform to diagnose malaria that can be used as a near patient tests (Point of Care, PoC). This platform combines PCR with a nucleic acid lateral flow immunoassay (NALFIA) to detect *Plasmodium* species in a multiplex format [21]. NALFIA is a simple read-out system, comparable to a dipstick, to visualise amplicons. The PCR-NALFIA system has an analytic sensitivity of 0.1 parasites/μL of blood (>100x more sensitive than conventional malaria diagnostics) with high sensitivity and specificity in clinical settings [21]. Importantly, the platform allows for direct amplification of *Plasmodium* DNA in whole blood [22] by utilising specific enzymes and buffers that are able to withstand the inhibitory effects usually encountered when directly amplifying DNA in blood. This avoids contamination-prone and labour-intensive parasite DNA extraction from clinical samples. This direct-on-blood (db)-PCR-NALFIA has passed rigorous laboratory evaluations and trials in disease endemic countries [22, 23]. Recently the dbPCR-NALFIA has been optimised by using a mini-PCR system (battery operated, reducing its dependency on electricity) thereby enhancing its field applicability. However, before this near point of care test can be recommended for routine practice, its performance needs to be evaluated in different clinical settings with differing malaria endemicities and health system contexts.

### Objectives {7}

The main objective of the project is to evaluate the diagnostic performance of the mini-dbPCR-NALFIA test for malaria in field settings. To achieve this objective the following specific project objectives will be achieved through a blend of clinical, bio-medical and socio-economic research.

#### Primary objective

To assess the diagnostic accuracy of the mini-dbPCR-NALFIA test for the diagnosis of malaria compared to routine diagnostic procedure(s) in place (microscopy and/or RDTs) and qPCR in different clinical and field settings: a high malaria incidence region (**Sudan**); a seasonal malaria transmission setting where HRP2-RDT may have false positive outcomes (**Burkina Faso**); an area with possible HRP2-deletions and high prevalence of *P*. *vivax* malaria (**Ethiopia**); a low transmission (near elimination) setting (**Namibia**); and **Kenya**, which has several different malaria zones: meso-hyperendemic (transmission all year round with seasonal peaks) in the lake and coastal areas; hypoendemic in the semi-arid regions, and potential for outbreaks in the highlands (non-immunes).

### Secondary objectives

1. To determine the direct and indirect costs and benefits of allocating limited resources to implement the mini-dbPCR-NALFIA test for malaria compared to current diagnostic strategies in place.
2. To identify health system characteristics likely to influence the introduction, implementation, and scale up of the new diagnostic tool (if cost-effective).

The study hypothesis is: “the mini-db-PCR-NALFIA will offer an improved diagnostic platform over microscopy and currently available HRP2-based RDTs for the detection of malaria infection in varied malaria epidemiological settings”.

### Trial design {8}

This will be a phase 3 diagnostic trial. Diagnostic performance will be assessed following the WHO/TDR guidelines for the evaluation of diagnostics [24] and the results will be reported according to STARD principles [25]. This phase 3 evaluation will be a prospective multi-centre diagnostic study on populations living in different malaria endemic settings and for which the disease status of each individual is not previously known [18]. Study participants (all ages) will be identified during their visits at the study health facilities. Subjects (or parent or legal guardian in case of minors) with suspected malaria will be asked to participate in the diagnostic study.

## Methods: Participants, interventions and outcomes

### Study setting {9}

The research will be carried out in 5 African countries (Burkina Faso, Sudan, Ethiopia, Namibia and Kenya), each representing an epidemiology of malaria different from the other:

- In **Burkina Faso**, the study will take place at the Clinical Research Unit of Nanoro (CRUN) trial site, located about 90 km from Ouagadougou. Malaria is hyper-endemic and transmission occurs throughout the year, with a peak during the rainy season (June–November). *P*. *falciparum* is responsible for >90% of all clinical malaria cases. Children under five years and pregnant women are the populations at highest risk. The total number of malaria cases for 2017 was >11 million. RDT remains the only diagnostic tool available to differentiate malaria from other infectious diseases in primary healthcare settings. The most common RDT used is *Pf*HRP2. A high rate of false positive RDT is reported which leads to misdiagnosis. A recent study showed that almost 50% of other causes of fevers were diagnosed as malaria due to false positive RDT results [9].
- Malaria transmission in **Sudan** is highly linked with climatic conditions. There are two transmission peaks; one during the rainy season (affecting most of Sudan) and the other during the winter season (notably in irrigation schemes and urban settings). In Sudan, 75% of the population is at risk. Malaria reported cases represent about 8.7% of total outpatient attendance and around 12.2% of hospital admissions. The main species is *P*. *falciparum* (87.6%). Diagnosis is made using microscopy and RDTs. The study will be performed in Gezira State under the supervision of the Blue Nile National Institute for Communicable Diseases (BNNICD)–University of Gezira in the outpatient clinics of three teaching hospitals and health centres with whom BNNICD has previously been conducting malaria research. These study sites may recruit more than 2,000 malaria cases per year.
- The study in **Ethiopia** will take place in Arba Minch (Southern Nations, Nationalities, and Peoples’ Region—SNNPR), Melka Werer (Afar) and Maksegnit (Gondar Zuria District). Arba Minch town (estimated population 174,980) is located 505 km south of Addis Ababa in Gamo Gofa zone with an average elevation of 1,209 m above sea level, an average annual temperature is 29.7°C and rainfall of 700 mm. Malaria is seasonal (September to December) and occurs in epidemic type where both *P*. *falciparum* and *P*. *vivax* are prevalent. There are two public health centres and one public hospital in Arba Minch town that provide healthcare services for the community in the town and surrounding areas. Around 179,386 febrile cases were attended to at these public healthcare facilities in Arba Minch in 2020 (MoH Report).
  - Maksegnit town (Gondar Zuria District) has one health centre and a malaria research unit. Gondar Zaria District is in North Gondar Zone, Amhara National Regional State of Ethiopia. Maksegnit is located at an altitude ranging between 1,107−3,022 meters above sea level, temperature ranging between 14–20°C (mean annual temperature of 17.9°C) and annual rainfall ranging between 1,030–1,223 mm (mean 1,100 mm). Both *P*. *falciparum* and *P*. *vivax* malaria are endemic.
  - Melka Werer (Amibara District) is located 265 km northeast of Addis Ababa in the Middle Awash valley. The climate of Amibara is generally semi-arid with temperatures ranging from 25°C−35°C and with an average annual rainfall below 600 mm. The altitude of Amibara lies between 720−1100 m above sea level. The 2019 population projection for Amibara District was 102,327. In Amibara District, there is one hospital (Mohammed Akle Memorial Hospital), three health centers (Worer, Sedefaga, and Awash Arba), 10 private clinics, and 17 health posts that provide health services to the population. Malaria is the top leading cause of morbidity in Amibara District. About 386,096 febrile illnesses and 15,0159 malaria cases were reported in 2020 by the health facilities.
- In **Kenya**, the study will be performed in two sites: one at the Lake Region (Busia County) which is endemic for malaria, and one in Kajiado County, a seasonal malaria area. At both sites 80% of the population are at risk; all malaria species occur but *P*. *falciparum* accounts for 98% of cases. Transmission is highest in May–July and January–March during the two rainy seasons. On a typical day, 50–100 patients with suspected malaria may be seen in a single health facility. Microscopy is the primary diagnostic approach in health facilities with laboratories, with RDTs also used for emergencies.
- In **Namibia**, the study will be conducted in an area that is classified as near elimination, the Zambezi region (North Eastern Namibia). The Zambezi region is primarily rural with most of the population engaged in subsistence farming. Malaria transmission is very heterogeneous in Namibia with transmission occurring during the rainy season peaking between January and May. The Zambezi region has accounted for more than 70% of the country’s malaria cases since 2020, the country at large had an incidence of 1.16 cases per 1000 in 2019. The country aims to eliminate malaria by 2022 and conducts case investigation of all malaria cases using Reactive Case Detection as malaria cases (both secondary and asymptomatic) have been shown to cluster around index cases. Reported malaria cases are almost all due to *Plasmodium falciparum*.

This selection of study sites also ensures a geographical coverage of Africa with partners from western, eastern and southern regions.

### Eligibility criteria {10}

For Sudan, Ethiopia, Burkina Faso and Kenya, the study will recruit participants who fulfil the following inclusion criteria:

1. Patients with clinically suspected malaria of all ages presenting at the health facility;
2. Coming from the health centre catchment area;
3. Informed consent from patients, or parents or guardians (in case of minors).

For Namibia, the study will recruit participants who fulfil the following criteria:

1. Residents of one of the selected households or have slept the night before in this household.
2. Informed consent from individual participants.

The exclusion criteria are not meeting the above criteria, or parents or guardians (in case of minors).

### Who will take informed consent? {26a}

Written informed consent will be obtained from all the study participants (or their legal representatives in case of minors) before enrolment. After an interview with the study clinician community health worker or other qualified member of the study team, the participant (or parent/legal guardian) will be asked to document their consent by signing an informed consent form; if the patient is a minor (according to applicable national law), the consent must be given by a parent or legal guardian. For minors in a specific age range informed assent is also obtained. Signed informed consent (or thumb-print whenever the participant or parents/guardians are illiterate) must be obtained before any blood sample or tests related to the study are carried out.

In addition, consent for the cost effectiveness study will be sought at the same time as enrolment to the diagnostic trial. Written informed consent will also be sought ahead of all structured interviews with key stakeholders for the health systems analysis.

### Additional consent provisions for collection and use of participant data and biological specimens {26b}

In the informed consenting procedure, participants and parent/guardians will be informed about future investigations on the collected blood samples. They will be informed that future investigations will only focus on understanding the performance and quality of the diagnostic procedures under study. For this purpose, part of the collected biological specimens may be transported and tested in Amsterdam (The Netherlands). No genetic testing about the human host will be performed. Some patient data will also be used for the health economics evaluation.

## Interventions

### Explanation for the choice of comparators {6b}

The diagnostic performance of the mini-dbPCR-NALFIA will be compared to the standard malaria diagnostic practices in place in the 5 participating countries (i.e. microscopy and/or rapid diagnostic test). Established qPCR will be used as the reference standard.

### Intervention description {11a}

Depending on the routine diagnostic procedures in place at each site, a rapid diagnostic test (RDT) or microscopy, or both, will be performed by the resident staff. RDT will be performed according to the manufacturers’ procedures. Thick and thin blood films will be prepared and Giemsa stained. Blood slides will be independently read by two expert microscopists. Parasite density will be calculated by counting the number of asexual parasites per 500 leucocytes in the thick blood film, based on an assumed WBC of 8,000 /μl by light microscopy at 1000 x magnification. In addition to routine diagnostic procedures in place, an additional blood sample (usually finger prick blood) will be collected for test evaluation (index and reference tests). Blood samples will be properly labelled with participants’ initials, inclusion number, protocol number, the study day and the date the sample is taken. For the index test (mini dbPCR-NALFIA) blood will be collected into an EDTA tube. For reference testing (qPCR), three blood spots will be collected on Whatman 3 filter paper [26]; one sample for qPCR, the second and third samples for QC procedures (10%) or back-up.

### Criteria for discontinuing or modifying allocated interventions {11b}

Only a single additional blood sample will be collected for the study. Treatment decisions will be made on the basis of routine diagnostic procedures in place. There will be no interference with standard diagnostic and treatment practices. There is therefore no need to define criteria for discontinuing or modifying interventions.

### Strategies to improve adherence to interventions {11c}

This study requires a single sampling point and there is no need for cases to be subjected to further interventions. Consequently, there is no need to design strategies to improve adherence to interventions.

### Relevant concomitant care permitted or prohibited during the trial {11d}

Not applicable, as this is a diagnostics study and only a single blood sample per study case will be tested. Prior use of anti-malarial drugs might influence diagnostic test performance, and prior drug use in the 14 days preceding collection of the blood sample will be recorded.

### Provisions for post-trial care {30}

As this is a diagnostic trial in which we are not testing any drug, such visits are not absolutely necessary. However, for ethical and safety reasons we will make a reasonable effort to contact all patients to check their health status and make sure they are recovering well. This visit can be done either physically or by telephone contact. Those who are not recovering well will be asked to return to the health facility for assessment and further management by the health facility staff and this action will be recorded in the CRF.

### Outcomes {12}

Outcomes will be reported according to STARD principles [25]. For each diagnostic test studied, we will report sensitivity, specificity, negative and positive predictive values (primary outcome). The outcomes of the evaluations will be further analysed using 2 x 2 tables (at 95% confidence intervals), and comparisons of the performance of the diagnostic test under investigation will be made with standard diagnostic procedures in place and qPCR as the accepted reference test to determine agreement between different tests (expressed as Cohen’s kappa values) [24].

### Participant timeline {13}

Participants will be asked to provide a clinical specimen and some demographic data on the day of enrolment. No further assessments or visits are planned in the context of this study

### Sample size {14}

The sample size for each country has been calculated according to the expected disease prevalence of the field sites to give adequate statistical definition to test sensitivity and specificity [24, 25] Sample size calculation is based on a 95% confidence interval using the equation proposed by Kiemde et al [9]: p +/-1.96 x √[p(1-p)/n] where p = sensitivity (or specificity) measured as a proportion and n = number of samples from infected people (or for specificity from non-infected samples).

Sample size calculations are further based on a conservative estimation of the number of actual cases amongst febrile patients (clinically suspected of having malaria) attending the various health facilities participating in the study. Furthermore, we have taken into account that there might be a withdrawal of consent of around 20% in each setting (most likely less). Based on these assumptions the following sample sizes have been determined for the following countries (note: there is no overall sample size calculation; each site has its own sample size and each is adequately powered; and the data can be analysed separately for each country):

- Sudan (10% incidence): 876 study cases;
- Ethiopia (10% incidence): 876 study cases;
- Burkina Faso (20% incidence): 438 study cases;
- Kenya (25% incidence): 351 study cases;
- Namibia: prevalence is <1.5% in the near elimination study region. prevalence is <1.5% in the near elimination study region. Therefore, participants will be recruited using passive and reactive case detection (RACD) targeting 2,000 people in the study areas as was also done in a previous study [27].

### Recruitment {15}

Prior to the start of the study, in some sites, communities and community leaders will be informed about the purpose of the study to enhance participation. All study sites have a sufficiently large population and malaria incidence to reach the projected sample size.

## Assignment of interventions: Allocation

### Sequence generation {16a}

Not applicable, as there will be no allocation to different study arms. All subjects will be subjected to the same procedure; i.e. collection of a blood sample for diagnostic testing.

### Concealment mechanism {16b}

Not applicable, as there will be no allocation to different study arms. All cases will receive the same intervention.

### Implementation {16c}

Not applicable, as there will be no allocation to different study arms. All cases will receive the same intervention.

## Assignment of interventions: Blinding

### Who will be blinded {17a}

There is no procedure for blinding as all participants will be following the same study procedure. Study staff executing the different diagnostic tests will be blinded from the results of other tests (.i.e. tests that they are not performing). The results of routine diagnostic testing in place at the study site will be used to make treatment decisions as per guidelines of the respective national Malaria Control Programmes.

### Procedure for unblinding if needed {17b}

There is no procedure for unblinding.

## Data collection and management

### Plans for assessment and collection of outcomes {18a}

Study staff will be adequately trained in execution of diagnostic tests performed in the framework of the study, data collection and data recording prior to the start of the trial. All data must be initially documented in the source documents, and then entered in the Case Record Form. For this study, an electronic database (developed on Open Clinica Software) will be used, which can be defined and entered remotely using the Internet. The CRF and database will allow identification of the study, site and patient; recording of the selection and inclusion of patients in the study; and recording of all data collected at each different study site.

### Plans to promote participant retention and complete follow-up {18b}

A singe sample collection is foreseen with no follow-up. Therefore, there is no need to develop a plan to promote participant retention or to complete follow-up.

### Data management {19}

Data management will be handled by the Institut de Recherche en Sciences de la Santé (IRSS) which has the required expertise in all components of data management of clinical trials and epidemiological studies. The data management office is located in the main campus of the Clinical Research Unit of Nanoro (CRUN). Data will be collected onto paper-based source documents before being captured into the electronic CRF developed in Open Clinica. Data will be entered online in all five sites and sent to the central server located at CRUN. The central data base will be managed at the CRUN by a data manager, who will run regular consistency checks and identify queries to be resolved by the local investigators in each country. This system will allow the rapid identification of potential problems. Data entry and review will be performed following the Data Entry Guidelines and the Data Management plan provided to the sites by the CRUN. Besides this central management, as mentioned above, a study monitor will visit each site at least once and check the information entered into the electronic CRF against the source documents available on site. Any modification made on the electronic CRF will be automatically registered. The final database will be obtained after resolution of all queries and will be locked for statistical analysis to be carried out according to a pre-established plan that will be developed by the study statistician in collaboration with the site investigators. The statistical analysis plan will specify how to deal with statistical issues such as missing data and protocol violations.

### Confidentiality {27}

Every possible measure will be taken to ensure that all personal data collected during the study will be appropriately protected. The collection of personal information will be restricted to meet the objectives of the project, and only data relevant for the execution of the project and interpretation of data will be collected. Collected information will be kept for a minimal time period, which is regulated by national or international law. All personal data collected during the study will be kept confidential. A numerical identifier and participants’ 3 initials will be assigned to each CFR and sample. None of the records contain individual name identification. All signed informed consent and assent forms and other personalised information will be kept under lock and key and will be available only to the project coordinator, principal investigators and data managers. Information stored in electronic databases will be protected by unique usernames and passwords, which will only be made available to appropriate authorised personnel. All records and data entry will be checked independently on-site by the research clinician and/or by the local PI to ensure accuracy. The collected information will never be transferred to countries lacking appropriate data protection or made available without restriction to third parties that are not directly linked to the project.

### Plans for collection, laboratory evaluation and storage of biological specimens for genetic or molecular analysis in this trial/future use {33}

Blood samples will be collected for routine malaria diagnostic tests and molecular analysis with PCR, which is used as the reference test for the diagnostic procedures followed in this trial. This work will be restricted to the assessment of the presence of *Plasmodium* species only. There will be no analysis of human genetic material. Collected specimens could be used for future diagnostic test development and evaluation, and if this will be the case, explicit informed consent from study participants will be obtained.

## Statistical methods

### Statistical methods for primary and secondary outcomes {20a}

The outcomes of the evaluations will be analysed using 2 x 2 tables (at 95% confidence intervals) and comparisons will be made with standard diagnostic procedures in place (microscopy and/or RDT) and qPCR as the accepted reference test to determine agreement between different tests (expressed as kappa values). Positive and negative predictive values will be calculated. Sensitivity and specificity of the db-PCR-NALFIA are assumed to be 95%.

### Interim analyses {21b}

None planned.

### Methods for additional analyses (e.g. subgroup analyses) {20b}

Next to the assessment of the diagnostic performance of the molecular platform, an economic evaluation and health systems analysis will be conducted to identify the incremental costs and benefits to healthcare providers and users of introducing and routinely using the mini-dbPCR-NALFIA for *P*. *falciparum* detection compared to routine diagnostic practices for malaria in the 5 different study localities (in Burkina Faso, Ethiopia, Kenya, Namibia and Sudan).

#### Cost effectiveness analysis (CEA)

The performance of the mini-dbPCR-NALFIA in terms of sensitivity and specificity will be combined with costs to the health system (this will include activities associated with the introduction, roll-out and scale-up of the mini-dbPCR-NALFIA within health facilities) to estimate the cost-effectiveness of the novel malaria diagnostic tool. The health outcomes will build on the Phase 3 trial primary endpoints and model the cost per disability-adjusted life years averted.

#### Health system analysis

Potential costs, barriers and facilitators associated with the implementation of the mini-dbPCR-NALFIA will be identified from both the supply and demand side and will imply some data collection alongside the main trial.

Supply Side—health system information on barriers and facilitators will include:

- Introduction / Start-up costs: Information will be gathered through document reviews and during key informant interviews at different health system levels (Ministry of Health/national, region/province/district and health facility) in order to understand which activities should be undertaken for the introduction of the mini-dbPCR-NALFIA and their associated resources and expected costs at each level of the health system (e.g., how many training courses would be needed for introducing the novel tool into routine malaria management?) In response to some of the key learnings of the recent Lancet Commission on Diagnostics [28], we will explore the broader diagnostic context in each of the 5 study countries to inform the introduction of a test such as NALFIA. Specifically, we will explore (i) the extent there is a formal (sub)national diagnostics strategy, (ii) if and how an evidence-based essential diagnostics list (EDL) is utilized and (iii) how diagnostic tools are prioritized and financed.
- Implementation / Running costs: Interviews will be conducted with key personnel at health facilities where mini-dbPCR-NALFIA will be used as part of the main study to help ascertain the costs and resources needed to routinely use dbPCR-NALFIA. These costs will largely reflect the cost of the tool and associated personnel costs for administration.
- Feasibility & Scale up: If the mini-dbPCR-NALFIA is cost effective, is it also affordable? How can the supply chain best accommodate this novel diagnostic tool? What would be the market barriers? What is the potential scope for private sector involvement in any scale up? This information will be gathered through document reviews and during key informant interviews in each of the five countries conducting the trial.

Demand side—recipients’ barriers/facilitators will be identified by collecting information from participants involved in the trial on:

- Costs of seeking diagnosis for fever or other symptoms suggestive of malaria (direct and indirect costs, provider choice, socio-economic characteristics).
- To understand the potential demand for a new product that is yet to reach the market, such as mini-dbPCR-NALFIA, we will ask patients their willingness to pay (WTP) for the product. WTP is a common economic approach that quantifies the hypothetical value potential users attribute to a product and its various characteristics, and the benefit they expect to obtain from it. Participants enrolled in the trial will be asked to participate in an economic method called the bidding game where they will be asked if they are willing to pay a base amount of money that will go up or down depending on the participants reply at each bid.
- The perception of risk from malaria may impact on the uptake of the mini-dbPCR-NALFIA and have implications for delivery strategies (e.g. facility-based versus community-based screening). A survey divided into perceptions of local health problems, malaria knowledge, prevention measures, and malaria risk perception will explore how study participants view the threat of malaria and how this may influence their perceptions of the near POC being trialled.

### Methods in analysis to handle protocol non-adherence and any statistical methods to handle missing data {20c}

Protocol non-adherence is not expected to occur as the trial only involves a single collection of a blood sample from the study cases. In case missing data occur, the whole data set of a participant will be removed from the analysis. This removal will be compensated by a slight over recruitment in number of study participants.

### Plans to give access to the full protocol, participant level-data and statistical code {31c}

The study has been registered in two accessible clinical trial registers and the information contained in these registers provide sufficient insights into the full trial design. The final results of the study will be published in peer-reviewed Open Access journals and presented at scientific meetings. None of the trial material may be disclosed to any party not directly involved in the study without written permission from the consortium partners. Presentation and publication of the trial results will be jointly carried out by the project investigators. Each African collaborator will be in charge of sharing relevant results with their respective health authorities, including the National Malaria Control Programmes. This will be done through ad-hoc meetings, data dissemination workshops and/or during Malaria Days in each country. Trial data will be accessible for inspection by appropriate health and regulatory authorities

## Oversight and monitoring

### Composition of the coordinating centre and trial steering committee {5d}

The trial is coordinated by dedicated trial staff at CRUN (Nanoro, Burkina Faso) and includes data management. CRUN is responsible for generic study protocol development and implementation. Each country is responsible for the implementation of the protocol in its respective sites, ethics review of the study protocol and other licensing requirements. An independent trial monitor has been appointed who will perform monitoring visits to all 5 study countries.

### Composition of the data monitoring committee, its role and reporting structure {21a}

CRUN (Burkina Faso) is responsible for centralised data management and monitoring. The central data base will be managed at CRUN by a dedicated data manager, who will run regular consistency checks and produce queries to be resolved by the local investigators. This system will allow the rapid identification of potential problems. Data entry and review will be performed following the Data Management Plan. Data management is independent from the sponsor.

### Adverse event reporting and harms {22}

The intervention is unlikely to result in (unexpected) adverse events or harms. An incidental findings policy is in place. This project will not involve research conducted towards genes and/or the genetic basis of diseases on human subjects. The research is not intended to provide subjects or their families with specific information about their genetic status. Furthermore, the research will be focused on the diagnosis of malaria only (a very common and well known condition in the study sites) and not on any other diseases or conditions, and does not actively search for human genetic disorders. Therefore, any other medical problems detected in patients with malaria or those testing negative for malaria will be managed appropriately within the health system. The risk that incidental findings occur in this study is very low though it might be the case when blood slides are examined by microscopy that there is a chance that other parasites, such as trypanosomes, may be found. In such cases the attending clinician will be informed to undertake appropriate medical actions.

### Frequency and plans for auditing trial conduct {23}

Each site will be visited by an independent monitor at least once during the conduct of the trial plus a study initiation visit at the start of study activities and a close-out visit after the last patient has completed the study procedures. The task of the Monitor is to verify the best conduct of the study through frequent contacts by telephone and in person with the Principal Investigator and site staff, in accordance with applicable regulations, Good Clinical Practice requirements and study-specific Standard Operating Procedures. The objectives and specific tasks of the Monitor are described in the ICH Guidelines E6. Source documents will be kept for each participant in the study. All information in the electronic CRF must be traceable to these source documents in each participant’s file. The Monitor, who is bound by a confidentiality agreement to protect participants’ confidentiality, has access to all relevant source documents to confirm their consistency with the electronic CRF entries.

### Plans for communicating important protocol amendments to relevant parties (e.g. trial participants, ethical committees) {25}

Protocol amendments will be communicated to the funder (EDCTP), sponsor (AMC) and relevant ethics review bodies in the country concerned.

### Dissemination plans {31a}

Dissemination of the study findings is planned through scientific papers and conference attendance, and also through meetings with various audiences of interest, including key stakeholders such as the health facilities involved, public health authorities and diagnostic companies who might be interested in bringing the diagnostic platform to market.

## Discussion

Among the factors that have contributed to the introduction of malaria RDTs into the health system in LMICs are the short running time, easy read-out system, limited infrastructural needs combined with their relatively low costs [29, 30]. Unfortunately, the diagnostic reliability of these RDTs still remains a challenge [31–34]. Malaria microscopy is still considered the accepted standard for the clinical diagnosis of *Plasmodium* infections, but requires qualified laboratory technicians, well maintained microscopes and a conventional laboratory. Microscopy also has a detection limit for low density infections and it is not accessible to many patients in endemic areas. This is even truer for diagnostics based on molecular biology, the most sensitive tools for the diagnosis of malaria, which needs specific equipment, sophisticated infrastructure and high-level trained laboratory personnel [35]. To circumvent this challenge in areas without laboratory facilities, the present study presents a diagnostic platform that combines direct-on-blood PCR amplification (circumventing DNA extraction and using simplified miniaturised battery operated equipment) with a simple NALFIA readout system to diagnose *Plasmodium* species in a multiplex format [21].

This study is designed towards the validation of the clinical performance of the mini-dbPCR-NALFIA for the detection of *Plasmodium* species in real life conditions. Based on previous studies [21, 22, 23], it is obvious that the proposed technology will improve diagnostic performance, as it is a highly sensitive tool applicable in specific settings that require improved tools for the diagnosis of malaria. These settings include regions where malaria diagnosis with RDT may lead to false positive results, where *hrp*2 deletion in *P*. *falciparum* leads to false negative results, or settings where sensitive diagnostics are needed to detect the last reservoir of parasite infection to achieve malaria elimination [6–8, 12, 13, 15, 16, 36]. If successful, this study will be able to provide necessary evidence to introduce the new diagnostic platform for the diagnosis of malaria which can be implemented particularly in settings where current diagnostics are inadequate due to sensitivity and/or specificity issues.

This evidence will be complemented with additional data on the costs associated with implementation, and information on patients and healthcare providers’ acceptance of the new diagnostic device. These activities will be implemented in parallel with the assessment of the new diagnostic platform. Despite successful evaluation of the diagnostic test, its integration within the health system may be compromised by patients’ and/or health staff’s low acceptability of the platform [37, 38]. This explorative study will be conducted to determine the acceptability of the new diagnostic platform by heath care providers as well as patients, and identify potential health system implementation bottlenecks in each country.

One of the major expected key deliverables of this project is to pave the route to marketing of the platform. Indeed, RDTs and microscopy will continue to be the backbone of clinical diagnosis in the near future, but performance limitations of RDTs mean that they cannot be used to screen populations for malaria infection in low transmission settings, for surveillance of populations as part of elimination programmes, in settings where RDTs have low specificity such as for treatment follow up, or in regions where HRP2-deleted parasites are becoming increasingly prevalent. It is into these market niches that the mini dbPCR-NALFIA fits very comfortably. For example, there are currently 18 countries with malaria elimination or eradication programmes in place. While these programmes vary in the number of tests carried out per year, the potential market size is estimated at millions of tests per year. The market for a sensitive screening diagnostic test will increase in future years as more countries introduce elimination and eradication programmes or will have measures in place to sustain their malaria free status.

The project may also face some operational challenges. Currently many research resources are allocated to combat the worldwide Covid-19 pandemic. Fortunately, our research sites are highly dedicated to perform malaria research, as this important infectious disease is still considered to have a huge impact on human health in the coming years. This is stressed by the fact that vaccines are not readily available and control measures are starting to lose their effectiveness due to emerging resistance to commonly used anti-malarial drugs. Furthermore, civil unrest is occurring in some of the participating countries. However, so far these have not affected our studies.

### Trial status

This is version 1.2, dated 2 July 2020, of the protocol. Ethics approvals have been obtained. Study staff are recruited and trained. At the time of submission, the recruitment of study participants started on 10 November 2021 in Kenya, on 7 December 2021 in Burkina Faso, 4 January 2022 in Sudan and 23 February 2022 in Namibia. Start in Ethiopia is pending. The trial is ongoing. Recruitment is expected to be completed 31 December 2022 and the final results of the study are expected end of 2023.

## Supporting information

S1 File Model consent form. (DOCX)

S2 File Ethical approvals. (DOCX)

## Abbreviations

AAU: Addis Ababa University
AMC: Academic Medical Centre
BNNICD: Blue Nile National Institute for Communicable Diseases
CE: certification
CMA: Centre Médical avec Antenne Chirurgicale
CRF: Case Report Form
CRUN: Clinical Research Unit of Nanoro
dbPCR-NALFIA: direct-on-blood PCR Nucleic Acid Lateral Flow ImmunoAssay
DBS: Dried Blood Spot
DNA: Deoxyribonucleic Acid
EDCTP: European & Developing Countries Clinical Trials Partnership
EDTA: ethylenediaminetetraacetic acid
EQAS: External Quality Assessment Scheme
GC/LP: Good Clinical and Laboratory Practice
HRP2: histidine-rich protein 2
IRSS: Institut de Recherche en Sciences de la Santé
LMICs: low and middle income countries
MDT: molecular diagnostic test
MoH: Ministry of Health
NALFIA: Nucleic Acid Lateral Flow Immunoassay
[n]PoC: near Point-of-Care
P. falciparum: *Plasmodium falciparum*
PCR: polymerase chain reaction
*Pf*HRP2: *Plasmodium falciparum* histidine-rich protein 2
*p*LDH: *Plasmodium* lactate dehydrogenase
PoC: Point-of-care
QC: Quality Control
qPCR: quantitative PCR
RBC: Red Blood Cell
RDT: Rapid Diagnostic Test
SOP: Standard Operating Procedure
SMC: seasonal malaria chemoprevention
SSA: sub-Saharan Africa
TDR: Special Programme for Research and Training in Tropical Diseases
WBC: white blood cell
WHO: World Health Organization
WP: Work Package
WTP: willingness to pay
