# Supplementary material for: Phase 3 evaluation of an innovative simple molecular test for the diagnosis of malaria in different endemic and health settings in sub-Saharan Africa (DIAGMAL)
Source: PLoS One. 2022 Sep 1;17(9):e0272847. doi: 10.1371/journal.pone.0272847 (PMC9436057; doi:10.1371/journal.pone.0272847)

S3 ETHICAL APPROVALS

**Ethical approval for Blue Nile National Institute for Communicable Diseases – University of Gezira, Sudan**


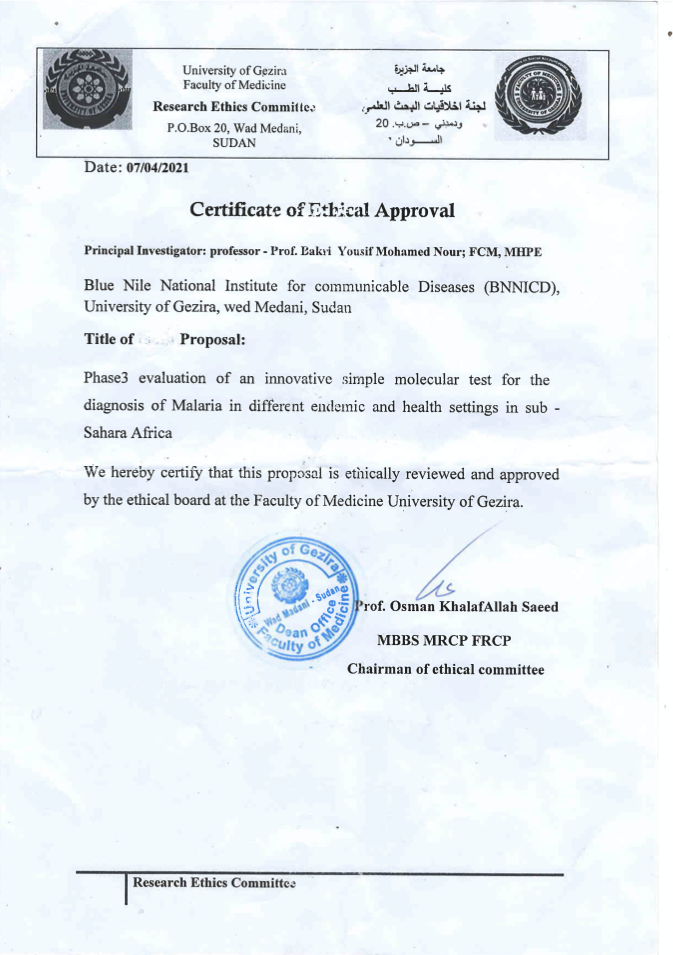


**Appendix C: Ethical approvals for IRSS-DRCO/Clinical Research Unit of Nanoro (CRUN), Burkina Faso**


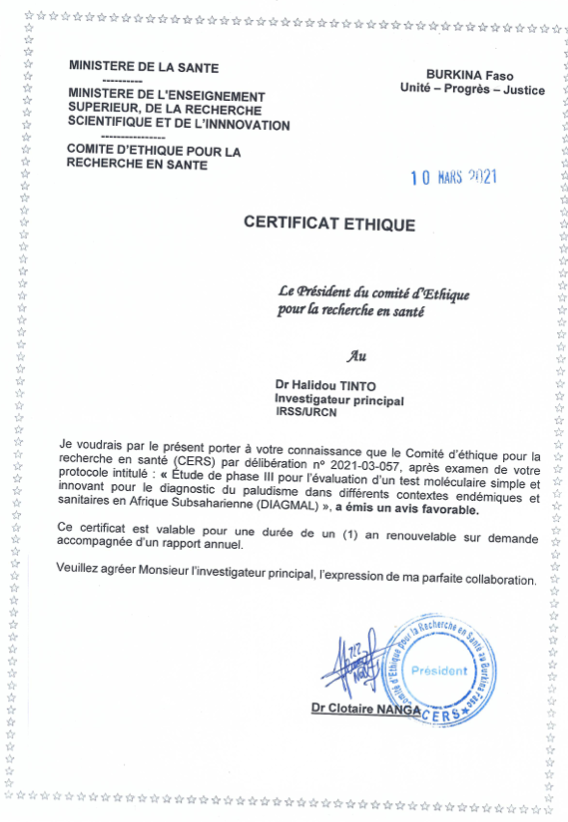


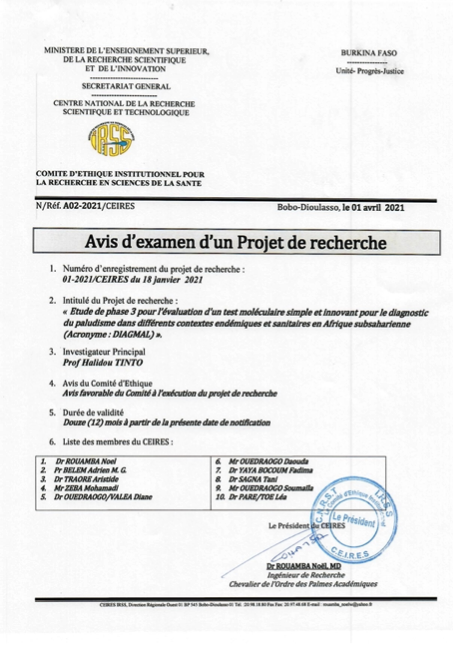


**Appendix D: Ethical approvals for Addis Ababa University (AAU), Ethiopia**


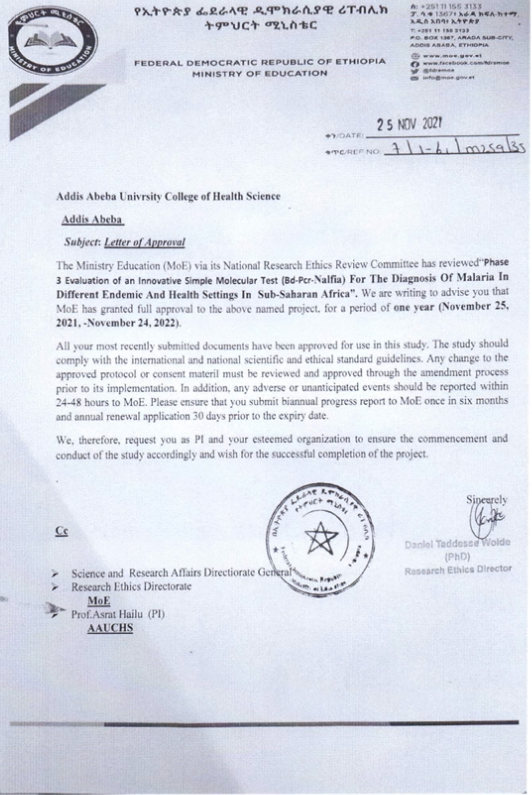


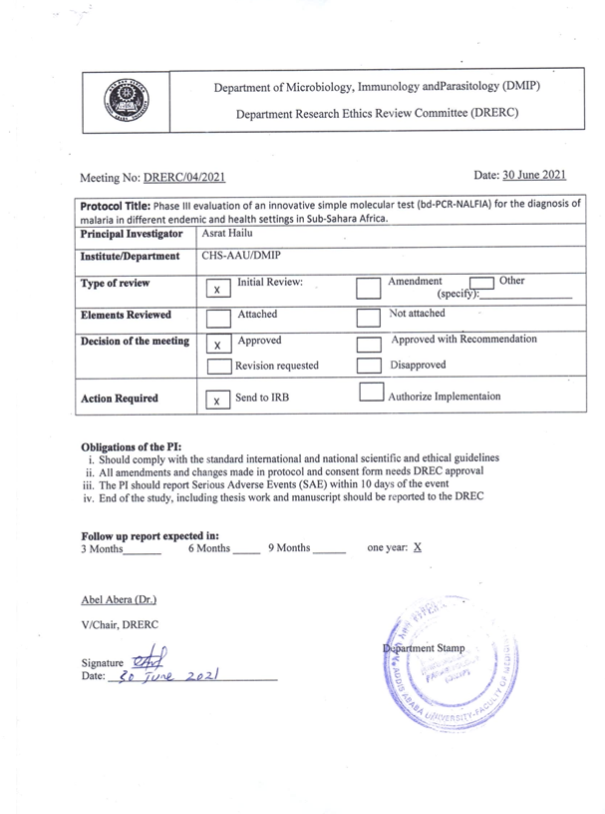


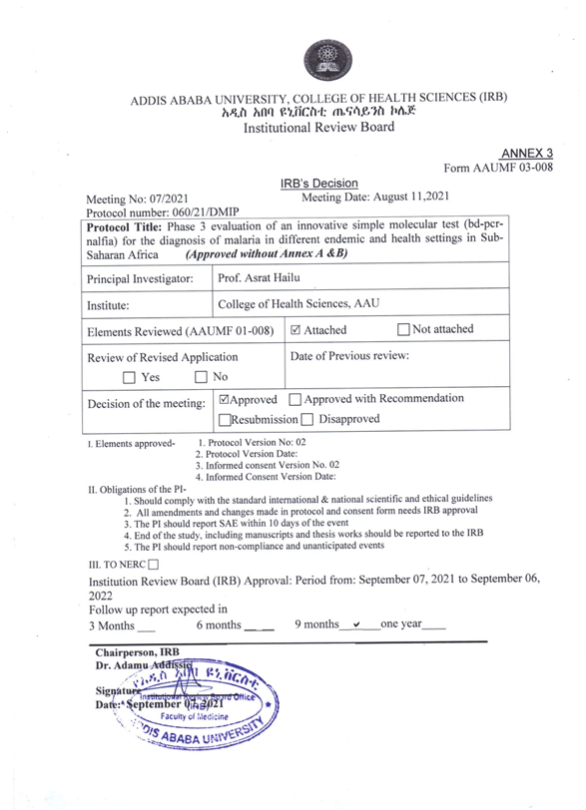


**Appendix E: Ethical approval for Amref Health Africa Headquarters, Kenya**


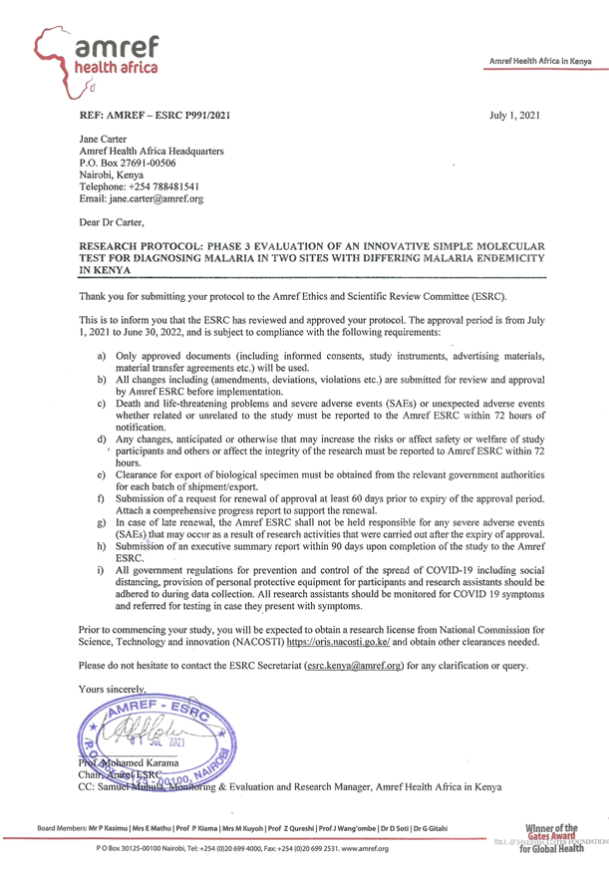


**Appendix F: Ethical approval for University of Namibia, Namibia**


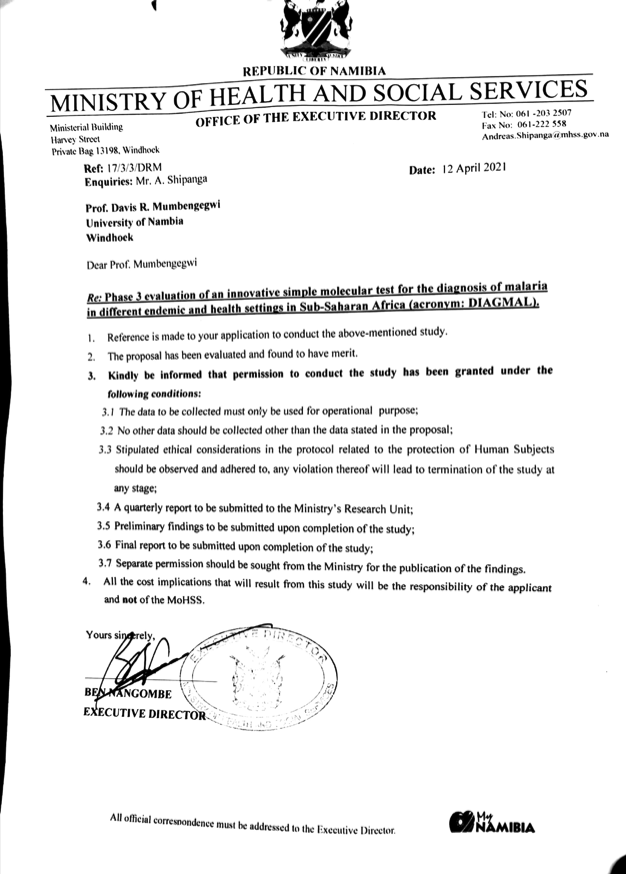

Supplement: S1 File — (DOCX) [file pone.0272847.s001.docx]
