# Supplementary material for: Phase 3 evaluation of an innovative simple molecular test for the diagnosis of malaria in different endemic and health settings in sub-Saharan Africa (DIAGMAL)
Source: PLoS One. 2022 Sep 1;17(9):e0272847. doi: 10.1371/journal.pone.0272847 (PMC9436057; doi:10.1371/journal.pone.0272847)
Supplement: S2 File — (DOCX) [file pone.0272847.s002.docx]

**S2 – MODEL CONSENT FORM**

**(This document should be adapted by each site)**

**RESEARCH PARTICIPANT INFORMED CONSENT FORM**

INFORMATION SHEET

STUDY TITLE

Phase 3 evaluation of an innovative simple molecular test for the diagnosis of malaria in different endemic and health settings in sub-Sahara Africa

Please read the background information and informed consent form carefully. The background information explains your rights and our responsibilities to you. If you have any questions concerning the study, please do not hesitate to ask the study doctors or nurses. Before you decide, it is important for you to understand why the research is being done and what it will involve. If you agree to participate in the study, you will be given a copy of this background information and of the signed document (Informed Consent Form) to take home with you.

**YOU MUST KEEP THIS BACKGROUND INFORMATION WITH YOU THROUGHOUT THE STUDY PERIOD.**

**PURPOSE OF THE STUDY**

Malaria is a sickness caused by a parasite that gets into the body when a mosquito bites you. It can cause fever, headache, body ache, and weakness. If it is not treated, it can make a person very ill.

This research study is being done to learn more about how malaria can be detected when it is in your blood. As you may know, there are many diseases including malaria that can give you fever. The only way to know the reason why you have fever is to seek in your blood the organism causing that fever. To know if your fever is due to malaria, we can take a drop of your blood and look at it in a machine called a microscope to seek the organism causing the disease. However, this machine requires electricity and a well-trained person to properly do the work. To solve these difficulties, the government introduced recently another tool using a simple test not requiring electricity that is called a Rapid Diagnostic Test (RDT). However, we have found out that sometimes this test is not able to detect malaria even if you have it, or it says that you have malaria when this is not true. In this context it is important to find other tests that can detect malaria better and more easily than the ones currently used.

The purpose of our research is to test a new machine that we think could be better than the microscopy and the RDT tests for detecting malaria.

This research will be done in 5 African countries: Burkina Faso, Ethiopia, Kenya, Namibia and Sudan. In Burkina Faso, the study will be carried out in the Nanoro Health District catchment area and a total of 438 patients suspected of having malaria will be recruited.

**HOW THE STUDY IS DONE**

If you agree to participate in this study, a blood test that is used routinely to check if you have malaria will be done immediately. In addition, we’ll take an additional blood sample to test our new machine and do other tests in the laboratory to compare with the results of the routine test done by the health facility.

At day 7 we’ll invite you or call you by phone to check if you are recovering well or not. If the malaria is not going away from your blood, a new treatment may be prescribed after re-examining you.

Some of the blood samples collected in the framework of this study will be sent to another laboratory in Amsterdam to check if the work we are doing is good or not (Quality Control).

**Risks**

The risks to the safety of participants in the study is negligible as we’ll not give you any drug. In addition, our new test will not influence the care the doctor will give you as he will treat you based on the results of the health facility’s usual tests. The only risk we see could be that related to the blood collection*.* Indeed, the risks of drawing blood from a finger prick include temporary discomfort from the needle stick, bruising, skin infection, and fainting. However the amount of blood collected is too small to affect your health, and any discomfort will rapidly go away.

**Benefits**

***For Sudan, Ethiopia, Burkina Faso and Kenya***: You will not have to pay the service fee for any of your clinic visits during this study. You will be closely followed until day 7 to make sure the treatment prescribed to you is working well.

This study will also help us learn how the new machine we are testing will work best in patients. This may help you or someone you know in the future.

There will be someone here at the clinic every day. You may come for a visit at any time if you feel that you are ill, even on nights or weekends or before the visit of day 7.

***For Namibia***: There is no cost to participate in this study. Also, you will not receive any payment for your participation. However, if we find that your child has malaria, we will provide free treatment to your child, according to national guidelines.

In addition, as it is possible that some members of your family may also have malaria, we will screen all those of your family who wish to come to the hospital to be tested if they also have the disease. A doctor from the research team will explain and advise you about the condition.

**Compensation**

You will not receive any compensation for your participation to this study. However, your costs for transport will be compensated.

**Confidentiality**

We will keep all information about you private to the extent allowed by the law. The specific information we learn about you will not be shared with anybody except the study investigators, your doctors, and will be written in your medical record. The finding of this study will be reported to the ministry of health in summary form. Your name will not appear on any reports from this study. The findings from this study may be published in a medical journal. The study participants will not be identified by name. After the study is completed, you may request an explanation of the study results.

**Contacts**

If you later have questions or concerns about your participation in this study, you may speak with one of our staff or contact the Study Coordinator at the following phone number: Tel: xxxxxxxxx If you have any questions about your rights, please contact the Ethics committee who has reviewed our protocol at the following phone number: Tel. xxxxxxxxx.

**Voluntary participation, refusal, and withdrawal**

It is important that you have been informed well about procedures and processes of taking part in this study:

- You are free to choose to be in this study or not. If you choose not to participate, you will still be treated and your medical benefits will not be affected.
- You may not receive money by taking part in the study. But knowledge may be gained that will benefit others.
- You may stop participating in the study at any time. This will not affect your medical benefits and you will still receive the usual diagnosis and treatment.

You do not have to be in this study to be treated for malaria. If you choose not to be in the study, you can still be treated for malaria at this hospital.

**Consent Form**

**Study Title:** Phase 3 evaluation of an innovative simple molecular test for the diagnosis of malaria in different endemic and health settings in sub-Sahara Africa

By signing or placing my thumbprint below, I am saying that:

***I have read this form, or it has been read to me; I have been able to ask questions about it, and my questions have been answered; and***

***I have been told that I have the right to stop participating in this study at any time without affecting my medical care.***

***I voluntarily agree to be in this research.***

_________ _________________________ _____________

Thumbprint - or - Signature Date

Name of legal guardian (if patient is a minor)

______ _________________________ _____________

Thumbprint - or - Signature Date

Name and role of the person obtaining the consent

Signature of person obtaining consent Date/Time

If the patient (or legal guardian) is unable to read and/or write, an impartial witness should be present during the informed consent discussion. After the written informed consent form is read and explained to the patient (or legal guardian) and after he/she has orally consented to his/her participation in the trial, and has provided his/her fingerprint, the witness should sign and personally date the consent form. By signing the consent form, the witness attests that the information in the consent form and any other written information was accurately explained to, and apparently understood by the patient (or legal guardian), and that informed consent was freely given by her.

Name of Person Witnessing Consent (printed)

Signature of Person Witnessing Consent Date/Time
